# Genetic Deletion of NR3A Accelerates Glutamatergic Synapse Maturation

**DOI:** 10.1371/journal.pone.0042327

**Published:** 2012-08-01

**Authors:** Maile A. Henson, Rylan S. Larsen, Shelikha N. Lawson, Isabel Pérez-Otaño, Nobuki Nakanishi, Stuart A. Lipton, Benjamin D. Philpot

**Affiliations:** 1 Curriculum in Neurobiology, University of North Carolina, Chapel Hill, North Carolina, United States of America; 2 Department of Cell and Molecular Physiology, University of North Carolina, Chapel Hill, North Carolina, United States of America; 3 Neuroscience Center, University of North Carolina, Chapel Hill, North Carolina, United States of America; 4 Carolina Institute for Developmental Disabilities, University of North Carolina, Chapel Hill, North Carolina, United States of America; 5 Cellular Neurobiology, Departamento de Neurociencias, Centro de Investigación Médica Aplicada (CIMA) y Universidad de Navarra, Pamplona, Spain; 6 Del E. Webb Center for Neuroscience, Aging and Stem Cell Research, Sanford-Burnham Medical Research Institute, La Jolla, California, United States of America; Institute for Interdisciplinary Neuroscience, France

## Abstract

Glutamatergic synapse maturation is critically dependent upon activation of NMDA-type glutamate receptors (NMDARs); however, the contributions of NR3A subunit-containing NMDARs to this process have only begun to be considered. Here we characterized the expression of NR3A in the developing mouse forebrain and examined the consequences of NR3A deletion on excitatory synapse maturation. We found that NR3A is expressed in many subcellular compartments, and during early development, NR3A subunits are particularly concentrated in the postsynaptic density (PSD). NR3A levels dramatically decline with age and are no longer enriched at PSDs in juveniles and adults. Genetic deletion of NR3A accelerates glutamatergic synaptic transmission, as measured by AMPAR-mediated postsynaptic currents recorded in hippocampal CA1. Consistent with the functional observations, we observed that the deletion of NR3A accelerated the expression of the glutamate receptor subunits NR1, NR2A, and GluR1 in the PSD in postnatal day (P) 8 mice. These data support the idea that glutamate receptors concentrate at synapses earlier in NR3A-knockout (NR3A-KO) mice. The precocious maturation of both AMPAR function and glutamate receptor expression are transient in NR3A-KO mice, as AMPAR currents and glutamate receptor protein levels are similar in NR3A-KO and wildtype mice by P16, an age when endogenous NR3A levels are normally declining. Taken together, our data support a model whereby NR3A negatively regulates the developmental stabilization of glutamate receptors involved in excitatory neurotransmission, synaptogenesis, and spine growth.

## Introduction

In early postnatal development, the formation and maturation of excitatory synapses play critical roles in the proper wiring of neuronal networks required for learning and memory. The balance between synapse stabilization and elimination is highly sensitive to changes in the complement of synaptic proteins. The subunit composition of NMDA- and AMPA-type glutamate receptors (NMDARs and AMPARs) is particularly important for defining ionotropic glutamate receptor-mediated synaptic transmission. Synaptic activity and sensory experience modify synaptic function, in part by promoting the transition between ‘immature’ and ‘mature’ forms of NMDARs (from predominantly NR2B- to NR2A-containing) in the postsynaptic density (PSD) and by the synaptic incorporation of AMPARs. These changes regulate the stabilization of the PSD, the subsequent decline in functional plasticity of the synapse, and the spine growth associated with synapse maturation [1].

NMDAR activation is crucial for synaptic strengthening and weakening [1], [2], processes that are pronounced during early life [3], [4] and instructive for proper brain development. NMDARs form through the assembly of NR2 (A–D) and NR3 (A–B) subunits with an obligatory NR1 dimer [5], also referred to as GluN1-GluN3B subunits. Most research in the mouse forebrain has concentrated on the canonical subtypes, NR2A and NR2B. Recent reports, however, have shown that the inclusion of NR3 subunits with NR1 and NR2 subunits alters NMDAR functions by reducing currents, lowering calcium permeability, and reducing block by magnesium [6], [7], [8], [9], [10], [11], [12], [13], [14]. Thus, unlike most NMDAR subunits, NR3A acts in a novel, dominant-negative manner to limit receptor function and the ability of synapses to strengthen [8], [14]. Interestingly, however, when expressed with NR1 alone, in the absence of NR2 subunits, NR3-NMDARs form a glycine-sensitive cation channel [15], [16], [17]; although these NR1/NR3 channels appear to be expressed in myelin rather than neurons [18].

Maximal NR3A expression coincides with a period during which many synapses are being formed, stabilized, or eliminated [19]. Like the NR2A and NR2B subunits, NR3A expression is developmentally regulated. However, its profile is unique, being highly expressed in early postnatal life and downregulating sharply into adulthood in humans, monkeys, and rodents [20]. This suggests that the regulation of NR3A expression is a common feature of brain development and that the function of NR3A is similar between mammalian species. Immunogold electron microscopy experiments in wildtype (WT) mice have shown that NR3A is normally absent from large synapses [14], suggesting that the presence of NR3A-containing NMDARs may serve to limit synapse growth and maturation. In support of this idea, loss- and gain-of-function studies in NR3A mutant mice have shown that spine number and synapse size are increased in the absence of NR3A [8] and reduced with the overexpression of NR3A [14]. Importantly, NR3A expression also appears to limit the expression of long-term potentiation, a form of synaptic plasticity, and memory consolidation [14].

Given the importance of NR3A for synaptic function and memory formation, here we sought to further investigate how NR3A regulates the transition from immature to mature synapses. We aimed to define the subcellular localization of NR3A-containing receptors at peak expression levels (∼P8 in mouse forebrain), and to establish the consequences of deleting NR3A on PSD proteins. We found that NR3A is synaptically targeted during early development, and this preferential expression at the PSD is lost with age. The genetic deletion of NR3A causes NMDARs and AMPARs to concentrate prematurely at synapses, and results in enhanced AMPAR currents in CA1 hippocampal pyramidal neurons. However, these measures in NR3A-KO mice return to WT control levels at an age when endogenous NR3A expression is normally declining (∼P16 in mice). Our results support a model by which NR3A expression inhibits glutamatergic synaptic transmission, thus providing a molecular brake to limit the premature development of forebrain synapse size, strength, and the ability to form lasting long-term memories during early development [14].

## Materials and Methods

### Ethics statement

Animal use in this study was approved by the Institutional Animal Care and Use Committee of the University of North Carolina at Chapel Hill (protocol #10-115.0) in accordance with the recommendations in the Guide for the Care and Use of Laboratory Animals of the National Institutes of Health. Homozygous WT and NR3A-KO offspring were selected from a mixed heterozygote colony and then maintained on a C57BL/6J background as separate homozygous breeding colonies. The generation and genotyping procedure of NR3A-KO mice have been previously described [8]. All mice were maintained on a 12:12 light/dark cycle and sacrificed according to approved protocol guidelines.

### Tissue collection

Brains were removed from mice at postnatal (P) days 8, 16, and >40 (range from P40-55). Forebrain tissue was rapidly dissected, frozen on dry ice, and stored at −80°C until use.

### Subcellular fractionation

One to three forebrains from each genotype, NR3A-KO or WT, were pooled for each fraction (n = 5–10 fractions/antibody for each age). Biochemical fractions were prepared as described [14], ending with the collection of the first detergent-extracted PSD fraction. Briefly, tissues were dounce-homogenized in HEPES-buffered sucrose (4 mM HEPES, 0.32 M sucrose, pH 7.4) and centrifuged (1000× *g* for 10 min) to yield the postnuclear supernatant (PNS) fraction. Synaptosomal pellets were lysed in hypo-osmotic solution, layered on a discontinuous gradient consisting of 0.32, 0.8, 1.0, and 1.2 M sucrose in 4 mM HEPES, pH 7.4, and subjected to density centrifugation (150,000× *g* for 2 h). The synaptic plasma membrane (SPM) fraction was collected from the 1.0/1.2 M sucrose interface, resuspended in 0.5% Triton X-100-containing buffer, and centrifuged to obtain the postsynaptic density (PSD) fraction. Complete protease inhibitors (Roche Applied Science, Germany), and phosphatase inhibitor mixtures 1 and 2 (Sigma-Aldrich, St Louis, MO) were added to all buffers. Procedures were performed on ice and/or in a cold room and fractions were stored at −80°C. Protein concentrations were determined by BCA Assay (Pierce Chemical, Rockford, IL). The following fractions were collected in this study: PNS, whole homogenate, postnuclear supernatant; CYT, cytosol; LM, light membranes; P2, crude synaptosomes; P3, lysed synaptic membranes; SPM, purified synaptic plasma membranes; TSF, Triton-soluble fraction; PSD, postsynaptic densities.

### Quantitative immunoblotting

Increasing amounts (1–15 µg) of total protein from each fraction were loaded in wells of 4–12% or 8% tris-glycine Novex gels (Invitrogen), resolved by SDS-PAGE, and transferred to nitrocellulose membranes. Blotting (Bio-Rad) and Odyssey system imaging and quantification (LI-COR) were carried out following manufacturers' protocols. The following antibodies were used at optimized concentrations: goat anti-NR1 (sc-1467, 0.01 µg/ml, Santa Cruz Biotechnology), rabbit anti-NR3A (07-356, 2 µg/ml, Millipore), goat anti-NR2B (sc-1469, 0.02 µg/ml, Santa Cruz Biotechnology), rabbit anti-NR2A (AB1555 and 04-901, 0.1 µg/ml, Millipore), rabbit anti-GluR1 (AB1504, 0.05 µg/ml, Millipore), mouse anti-PSD-95 (MAB1596, 1 µg/ml, Chemicon), mouse anti-synaptophysin (Syp, S-5768, 0.5 µg/ml, Sigma-Aldrich), mouse anti-β-tubulin (MAB 3408, 10 µg/ml, Chemicon), Alexa Fluor 680-labeled donkey anti-goat IgG (#A21084, Invitrogen), Alexa Fluor 680-labeled goat anti-mouse IgG (#A21058, Invitrogen), and IRDye 800-labeled donkey anti-rabbit IgG (#611-732-127, Rockland Immunochemicals). All samples were run multiple times.

### Hippocampal slice preparation

Mice were anesthetized with sodium pentobarbital and euthanized upon disappearance of corneal reflexes. Brains were rapidly removed and immersed in ice-cold dissection buffer (composition in mM: NaCl, 87; KCl, 2.5; NaH_2_PO_4_, 1.25; NaHCO_3_, 26; sucrose, 75; d-(+)-glucose, 10; ascorbic acid, 1.3; MgCl_2_, 7; and CaCl_2_, 0.5) and bubbled with 95% O_2_ and 5% CO_2_. The hippocampus was dissected and then 350 µm coronal slices were prepared using a vibrating microtome (VT1200S; Leica, Bannockburn, IL). Slices were allowed to recover for 20 min in a 35°C submersion chamber filled with oxygenated artificial cerebrospinal fluid (ACSF) (composition in mM: NaCl, 124; KCl, 3; Na_2_PO_4_,1.25; NaHCO_3_, 26; MgCl_2_, 1; CaCl_2_, 2; d-(+)-glucose, 20) and then kept at room temperature for at least 40 min until use.

### Voltage-clamp recordings

Patch pipettes were pulled from thick-walled borosilicate glass. Open tip resistances were 2–7 MΩ when pipettes were filled with an internal solution containing (in mM): cesium hydroxide, 107; d-gluconic acid, 107; TEA-chloride, 5; NaCl, 3.7; HEPES, 20; sodium guanosine triphosphate, 0.3; magnesium adenosine triphosphate, 4; EGTA, 0.2; BAPTA, 10; and QX-314 chloride, 5 (Alomone Labs), with pH adjusted to 7.2 and osmolarity adjusted to 300 mOsm by the addition of sucrose. Hippocampal CA1 pyramidal cells were voltage-clamped in the whole-cell configuration using a patch-clamp amplifier (Multiclamp 700A; Molecular Devices, Sunnyvale, CA), and data were acquired and analyzed using pCLAMP 9.2 software (Molecular Devices). Changes in series resistance were monitored throughout the experiment by giving a test pulse and measuring the amplitude of the capacitive current. Only cells with series resistance <18 MΩ were included for analysis. Input resistance was monitored throughout the experiment by measuring the amplitude of the steady-state current, filtered at 2 kHz, evoked from a test pulse. Only cells with <30% change in R_input_ and R_series_ were included for analysis. EPSCs were evoked from a stimulating electrode (two-conductor cluster electrodes with 75 µm tip separation; FHC, Bowdoin, ME) by stimulation (200 µs every 15 sec) of the stratum radiatum. For AMPA input-output curves, EPSCs were recorded at −80 mV and two trials at the same stimulus intensity were averaged to produce a measurement of AMPAR current at a given stimulus intensity. AMPAR currents were pharmacologically isolated by modifying the standard ACSF to contain picrotoxin (50 µM, Sigma-Aldrich) and d,l-APV (10 µM, Ascent Scientific), and this bath solution was perfused at 2 ml/min at 30°C. A minimum of three animals were used per experimental group.

### Data Analysis

Calculations of signal intensity per microgram protein were determined from multiple wells on each gel for each target antigen and then averaged across multiple gels. Fraction means per genotype were either presented as immunoreactive (IR) units/µg protein or normalized to mean control values and expressed as % of control or % of maximum. Error bars represent mean ± SEM. Statistical evaluations were performed using either two-tailed student's *t*-tests or one-way analyses of variance (ANOVAs) (for multiple group comparisons), followed by between-group comparisons with Tukey-Kramer tests (Graphpad Instat, San Diego, CA). Significance was placed at *p*<0.05.

## Results

### Biochemical enrichment of synaptic proteins by subcellular fractionation

**Figure 1:**
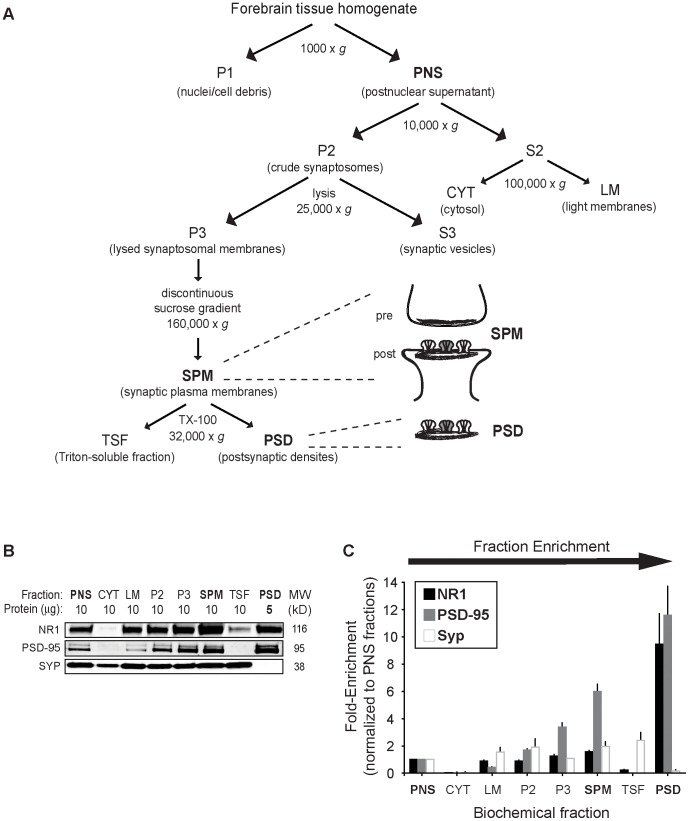
Subcellular fractionation of presynaptic and postsynaptic proteins in forebrain neurons. (**A**) Schematic illustration of the enrichment process, highlighting the key fractions examined in this study (see Methods for details). The PNS fraction obtained from the homogenate is subjected to a series of centrifugation steps to isolate pre- and postsynaptic plasma membranes (SPM), including layering on a sucrose gradient, and finally extracting with detergent to obtain the postsynaptic densities (PSD). (**B**) Representative immunoblots show NR1, PSD-95, and synaptophysin (Syp) expression in fractions from P16 mouse forebrain, with 5 or 10 µg total protein loaded in each lane as indicated. (**C**) Quantification of fraction fold-enrichment made with sample values normalized to PNS fractions. Error bars represent SEM. PNS, postnuclear supernatant; CYT, cytosol; LM, light membranes; P2, crude synaptosomes; P3, lysed synaptic membranes; SPM, purified synaptic plasma membranes; TSF, Triton-soluble fraction; PSD, postsynaptic densities.

To examine the subcellular localization of glutamate receptor subunit proteins in forebrain neurons, we performed immunoblot analysis on various fractions extracted from WT and NR3A-KO mice (**Figure 1a**), including selectively enriched homogenates for synaptic complexes (SPM) and postsynaptic densities (PSD). To demonstrate the quality of fractions prepared from homogenates derived from centrifugal removal of nuclei (PNS, postnuclear supernatant), the fractions were probed by immunoblot analysis of presynaptic and postsynaptic markers. The fidelity of this biochemical fractionation was validated by the near absence of membrane proteins (e.g., glutamate receptor subunit, NR1) in the cytosolic (CYT) fraction; negligible expression of the presynaptic protein, synaptophysin (Syp), in the PSD; absence of the postsynaptic density protein, PSD-95, from the Triton-soluble fraction (TSF); and progressive enrichment of NR1 and PSD-95 from the initial homogenate (PNS) to the PSD (**Figure 1b, c**). Because the PSD fractions are highly enriched, a two-fold reduction in protein loading over the previous fractions was used to avoid saturation of the blotted bands.

### NR3A is concentrated in neonatal PSDs and undergoes a developmental decline

**Figure 2:**
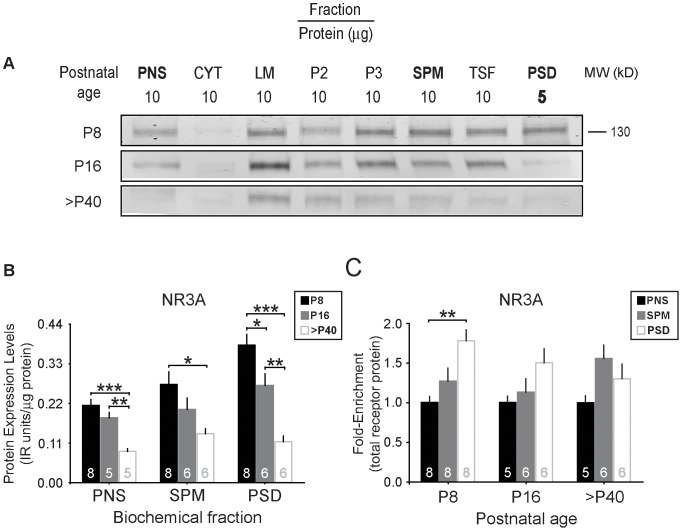
Developmental reduction in NR3A levels and PSD targeting. (**A**) Representative immunoblots of biochemical fractionation from P8, P16, and >P40 forebrain. (**B**) NR3A protein levels in the PNS, SPM, and PSD fractions from mice at P8, P16, and >P40. NR3A levels decrease during development in all fractions. Data are averaged means of immunoreactive (IR) values relative to protein loads (µg). (**C**) Averaged data normalized to initial homogenate values (total receptor protein) to highlight the shift in NR3A expression away from the PSD fraction. Error bars represent SEM. * *p*<0.05, ** *p*<0.01, *** *p*<0.001.

To determine the subcellular localization of NR3A-containing receptors over mouse forebrain development, we first determined to which biochemical compartments NR3A is targeted at its age (P8) of peak expression [21]. Western blots probed with an anti-NR3A antibody revealed that at P8, NR3A is expressed predominantly in membranous fractions, including the light membranes (LM) fraction that contains intracellular organelles (e.g., microsomes, endosomes, Golgi, endoplasmic reticulum), the synaptic junction (SPM) fraction containing pre- and postsynaptic proteins, and the PSD fraction (**Figure 2a**). These data are consistent with a previous report using juvenile rats [21]. The strong presence of NR3A in the different membrane fractions suggests that these receptors are mobile in the plasma membrane, as has been suggested of NMDARs in general [22].

Because NMDAR subunit composition and localization determines synaptic functions, we further probed how the subcellular expression of NR3A changes with age. Our study focused on the PNS, SPM, and PSD fractions taken from P8 (neonatal), P16 (juvenile), and age >P40 (young adult) mice. We found that NR3A levels decrease 50–70% from P8 through adulthood in all fractions (**Figure 2b**, PNS, F_(2,17)_ = 17.988, *p* = 0.0001; SPM, F_(2,19)_ = 5.397, *p* = 0.0153; PSD, F_(2,19)_ = 23.612, *p*<0.0001). The developmental profile we observed for NR3A is similar to that reported previously [20]. We found that NR3A is particularly enriched within the PSD at P8, when NR3A levels are highest [8], [21] (**Figure 2c**, P8, F_(2,23)_ = 8.973, *p* = 0.0015). However, NR3A levels in PSD fractions decrease significantly at juvenile and young adult ages (**Figure 2b**, PSD, F_(2,19)_ = 23.612, *p*<0.0001; P16_% of maximum_ = 70.8±8.4, *p*<0.05; >P40_% of maximum_ = 32.9±3.1, *p*<0.0001) and PSD enrichment is lost after P8 (**Figure 2c**, P16, F_(2,16)_ = 2.682, *p* = 0.1033; >P40, F_(2,16)_ = 3.034, *p* = 0.080). These data indicate that not only do NR3A levels decline dramatically during development, but also the privileged access of NR3A-containing NMDARs to the PSD is lost with age.

### Glutamate receptor proteins are enriched in PSD fractions

NR3A is thought to suppress NMDAR activity during early life to limit synaptic strengthening and stabilization [14]. As such, we hypothesized that manipulations of NR3A would alter the synaptic recruitment of glutamate receptor subunits involved in the maturation of excitatory synapses. Consistent with this hypothesis, we recently showed that the synaptic concentration of NR1 is significantly increased in neonatal NR3A knockout mice [14]. We reasoned that if NR3A negatively regulates NR1 localization and numbers at synaptic junctions, other glutamate receptor subunits might be similarly affected. To test how NR3A loss affects ionotropic receptor subunit expression, we first had to establish the normal developmental expression of selected subunits in our system. We chose to examine expression of NR1, NR2A, NR2B, and GluR1 subunits due to their high expression levels in developing rodent forebrain and their involvement in synaptic plasticity and maturation (mRNA: [23], [24], [25], [26], [27], [28], [29]; protein: [29], [30], [31], [32], [33], [34], [35], [36], [37], [38], [39], [48]).

**Figure 3:**
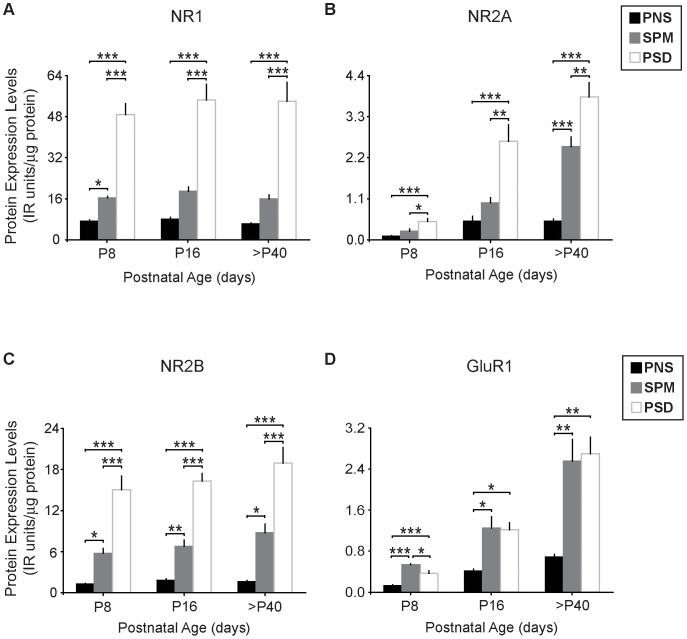
Glutamate receptor subunits NR1, NR2A, NR2B, and GluR1 are highly enriched in PSDs of postnatal mice. Relative protein levels of (**A**) NR1, (**B**) NR2A, (**C**) NR2B, and (**D**) GluR1 in mouse forebrain. Protein data are averaged means of immunoreactive (IR) values relative to total protein loads (µg). n = 5–9. Error bars represent SEM. * *p*<0.05, ** *p*<0.01, *** *p*<0.001.

We delineated the developmental expression profiles of these NMDAR and AMPAR subunits present in forebrain fractions from P8, P16, and young adult mice. This not only provided a baseline to which we could directly compare the NR3A data, but also confirmed the reproducibility of our biochemical fractionation protocol. Importantly, while the developmental profiles we observe for NR1, NR2A, NR2B, and GluR1 are generally consistent with previous reports, our analyses provide insights into the localization of these glutamate receptor subunits in different biochemical fractions across several ages (**Figure 3**).

Because NR1 is required for all functional NMDARs [40], [41], [42], we confirmed that NR1 levels are enriched from the initial PNS fraction to the PSD [8], [14], [21], [34], [37], [43] at each age and are similar across development [25], [34], [35], [36], [37], [39], [44] within each fraction (**Figure 3a**: NR1 comparison by ages: P8, F_(2,23)_ = 74.691, *p*<0.0001; P16, F_(2,17)_ = 44.507, *p*<0.0001; >P40, F_(2,16)_ = 29.234, *p*<0.0001; NR1 comparison by fractions: PNS, F_(2,18)_ = 1.636, *p* = 0.2258; SPM, F_(2,19)_ = 1.404, *p* = 0.2727; PSD, F_(2,19)_ = 0.3277, *p* = 0.7250.).

Similarly, NR2B showed abundant expression during early life and minimal change with age [25], [33], [36], [37], [44], [45] within each fraction, being highly enriched in the PSD compared to PNS homogenate fractions [21], [34], [37], [43], [46] (**Figure 3c**: NR2B comparison by ages: P8, F_(2,23)_ = 32.582, *p*<0.0001; P16, F_(2,17)_ = 77.483, *p*<0.0001; >P40, F_(2,17)_ = 31.964, *p*<0.0001; NR2B comparison by fractions: PNS, F_(2,19)_ = 2.304, *p* = 0.1302; SPM, F_(2,19)_ = 2.617, *p* = 0.1021; PSD, F_(2,19)_ = 1.046, *p* = 0.3730).

In contrast, we found that NR2A levels are very low during early development and increase dramatically after the first week of life, as expected [23], [25], [31], [33], [34], [35], [36], [37], [39], [44], [45]. This subunit was also concentrated in the PSD [21], [37], [43], [46] (**Figure 3b**: NR2A comparison by ages: P8, F_(2,22)_ = 11.725, *p* = 0.0004; P16, F_(2,16)_ = 14.377, *p* = 0.0004; >P40, F_(2,17)_ = 39.610, *p*<0.0001; NR2A comparison by fractions: PNS, F_(2,18)_ = 12.549, *p* = 0.0005; SPM, F_(2,19)_ = 53.233, *p*<0.0001; PSD, F_(2,18)_ = 28.548, *p*<0.0001). These increases in protein levels indicate that NR2A is significantly regulated across developmental ages, consistent with previous observations [29], [33], [34], [36], [37], [44], [45], [47], [48]. The developmental shift in NR2A and NR2B subunit composition (compare **Figure 3b** to **3c**) has been well-characterized, as it alters the ability of synapses to strengthen and weaken in response to activity [2]. Although we might have expected to see more enrichment of NR2A at PSDs when compared to NR2B, especially at adult stages (P8: 5- vs. 12-fold enrichment, respectively; P16: 5- vs. 9-fold enrichment; >P40: 8- vs. 12-fold enrichment), the high concentration of these subunits at the postsynaptic density is consistent with previous reports [34], [39], [46], and suggests that receptors containing NR2A and NR2B are preferentially targeted to the PSD.

In line with other studies [32], [38], [44], [49], [50], [51], we found that GluR1, like NR2A, also exhibits expression onset just after the first postnatal week, increases dramatically into adulthood, and is enriched in the PSD at all ages (**Figure 3d**: GluR1 comparison by ages: P8, F_(2,21)_ = 30.729, *p*<0.0001; P16, F_(2,15)_ = 6.454, *p* = 0.0113; >P40, F_(2,17)_ = 12.991, *p* = 0.0005; GluR1 comparison by fractions: PNS, F_(2,17)_ = 57.918, *p*<0.0001; SPM, F_(2,17)_ = 13.702, *p* = 0.0004; PSD, F_(2,19)_ = 40.306, *p*<0.0001). The preferential activity-dependent recruitment of GluR1-containing AMPARs is thought to result from LTP during the early phases of synapse maturation [52], [53], [54], [55].

### Synapse maturation markers, NR1, NR2A and GluR1, concentrate earlier in PSDs of NR3A-KO mice

**Figure 4:**
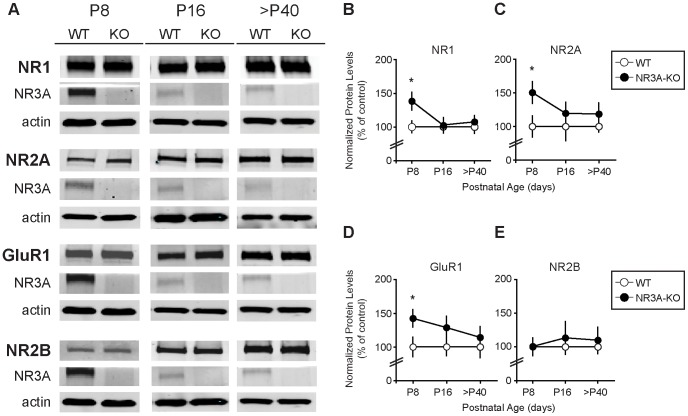
Deletion of NR3A transiently accelerates expression of synapse maturation markers. (**A**) Representative immunoblots from NR3A-KO compared to WT controls show increased PSD levels of (**B**) NR1, (**C**) NR2A, and (**D**) GluR1 at P8 that return to WT levels by P16 and adult ages. (**E**) NR2B expression is unchanged in the NR3A-KO. (**B, C, D, E**) Data are averaged means of immunoreactive values relative to protein loads (µg) and presented as percent of control values. NR1 values for age P8 are re-plotted here from [14] for comparative purposes. Error bars represent SEM. n = 7–10. Significance from control: * *p*<0.05.

To examine the effects of genetic deletion of NR3A on the composition of glutamate receptor subunits, we measured changes in protein levels of synapse maturation markers in NR3A-KO and WT mice over development. We found that both NMDAR and AMPAR subunit abundance in PSD fractions are significantly increased in P8 knockouts. Immunoblot analysis of synaptic membranes revealed that, like NR1 (**Figure 4a**, NR1_% of control_ = 138.6±13.4, n = 8–9, *p* = 0.033; data re-plotted from [14] for comparative purposes), PSD levels of synapse maturation markers, NR2A and GluR1, are also enhanced in NR3A-KO compared to WT mice at age P8 (**Figure 4b**, NR2A_% of control_ = 150.4±16.3, n = 7–8, *p* = 0.047; **Figure 4c**; GluR1_% of control_ = 142.4±12.9, n = 8, *p* = 0.046). Both NR2A and GluR1 WT expression levels are very low at this age (**Figure 3b**, NR2A_% of maximum_ = 12.69±2.02; **Figure 3d**, GluR1 _% of maximum_ = 13.49±1.95), and their increased levels in mutant PSDs indicate that the loss of NR3A promotes the early concentrations at synapses of both NR2A-NMDARs and GluR1-AMPARs. No differences were observed in total receptor protein fractions (PNS, data not shown). These data also indicate that NR3A limits synaptic expression of AMPARs as well as NMDARs. The increase of NR2A in the NR3A-KO PSD fraction may point to a critical role of NR3A in preventing NR2A from being targeted to the PSD.

Because the NR2B subunit is also highly expressed in immature forebrains during the early postnatal period, we examined whether any changes occurred for NR2B subunit expression in the NR3A-KO forebrain. However, in contrast to the pronounced upregulation of NR1, NR2A, and GluR1, we observed no discernible changes in NR2B expression levels (**Figure 4d**, NR2B_% of control_ = 100.6±9.3, n = 8–9, *p* = 0.972).

### Early onset of synaptic glutamate receptor concentration is transient

To determine whether the precocious concentration of glutamate receptor subunits in NR3A-KO mice is transient or sustained, we next examined mice at P16 and P40–55, the period during and after which synaptic refinements are completed, and when endogenous NR3A levels are declining. Interestingly, we found no significant differences between NR3A-KO and WT mice at P16 for NR1, NR2A, and GluR1 protein levels (**Figure 4**, NR1_% of control_ = 102.8±11.6, n = 10, *p* = 0.844; NR2A_% of control_ = 119.5±16.8, n = 10, *p* = 0.480; GluR1_% of control_ = 128.7±17.1, n = 10, *p* = 0.201). Although there appears to be a trend towards increased levels at older ages, this effect did not achieve statistical significance. The normalizationof the phenotype was maintained in more mature mice (**Figure 4**, NR1_% of control_ = 107.4±9.82, n = 9–10, *p* = 0.601; NR2A_% of control_ = 140.7±20.4, n = 10, *p* = 0.123; GluR1_% of control_ = 124.8±16.1, n = 10, *p* = 0.364), further confirming that the premature concentration of synapse maturation markers, NR1, NR2A, and GluR1 in the absence of NR3A is a transient effect. Again, changes in NR2B levels were not detected (**Figure 4**, NR2B_% of P16 control_ = 113.3±24.4, n = 9, *p* = 0.621 and NR2B_% of >P40 control_ = 87.1±17.6, n = 7–10, *p* = 0.583). The accelerated expression of NMDAR and AMPAR subunits in immature NR3A-KO mice, along with the strong presence of NR3A proteins in the developing but not adult rodent brain, support a model in which NR3A negatively regulates glutamate receptor expression during normal postnatal CNS development.

### AMPAR-mediated currents are also transiently enhanced and accelerated in NR3A-KO mice

**Figure 5:**
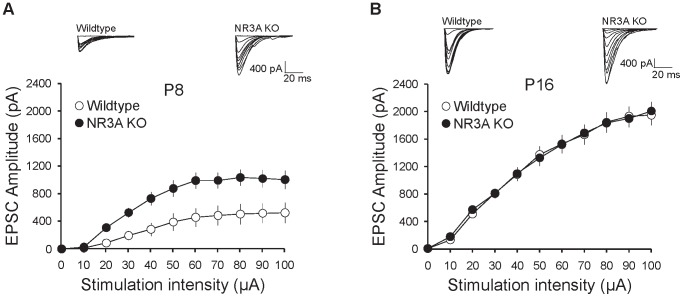
Genetic deletion of NR3A accelerates the expression of AMPAR-mediated currents. (**A**) AMPA input-output (I–O) curve demonstrating the amplitude of synaptic AMPAR currents recorded at −80 mV in CA1 pyramidal neurons from P8 NR3A-KO and WT controls (WT, n = 12 neurons; NR3A-KO, n = 12 neurons). RMANOVA revealed a significant effect of genotype on AMPAR current amplitude across stimulation intensities (F_(1, 10)_ = 9.557, *p* = 0.005). (**B**) Synaptic AMPA currents recorded at P16–17 are similar between WT and NR3A-KO mice (WT, n = 13 neurons; NR3A-KO, n = 11 neurons). In contrast to AMPA currents recorded at P8, there is no significant effect of genotype on synaptic AMPAR currents at this age (RMANOVA, *p* = 0.99). Data are averaged means of NR3A-KO and control values. Error bars represent SEM. Significance from control: * *p*<0.05.

In postnatal development, AMPARs are recruited to synapses following LTP, resulting in the structural and functional maturation of glutamatergic synapses [52], [53], [54], [55]. Since NR3A-KO mice demonstrate an early onset of hippocampal LTP [14] and GluR1 is prematurely upregulated in PSD fractions, we next used direct electrophysiological recordings to determine if functional AMPAR responses were also prematurely enhanced through development. To assay this, we recorded AMPAR currents from CA1 pyramidal neurons in the hippocampus. We found that AMPAR-mediated currents from P8 NR3A-KO mice displayed a two-fold increase in the amplitudes of evoked EPSCs, compared to their WT controls (**Figure 5a**). Similar to the biochemical results, the EPSC amplitudes from NR3A-KOs were indistinguishable from controls by P16 (**Figure 5b**). The early enhancement of AMPAR currents at P8 is in agreement with the early onset of LTP in NR3A-KO mice [14]. This indicates that the premature enhancements of both AMPAR function and protein expression are transient in NR3A-KO mice. Our observations thus support a model whereby NR3A limits NMDAR functionality and, by limiting downstream NMDAR signaling, NR3A-containing receptors reduce LTP-like insertion of GluR1-containing AMPARs during early development.

## Discussion

Inclusion of NR3A in NMDARs reduces currents, lowers calcium permeability, and lowers the sensitivity of NMDARs to magnesium block. Given the importance of NR3A in shaping NMDAR functions, it is surprising that relatively little is known about the role of this subunit at the synapse. Therefore, we tested the hypotheses that (1) NR3A-containing NMDARs shift from a synaptic to a peri-/extrasynaptic location with development, and (2) NR3A suppresses glutamate receptor activity during early life to prevent premature synaptic strengthening and stabilization. Our data show that NR3A is expressed at high levels and enriched within the postsynaptic density (PSD) during early postnatal life (P8). However, NR3A levels undergo a striking age-dependent decline and are no longer enriched in the PSD fraction after P8. Glutamate receptor subunit levels are increased in PSDs from neonatal NR3A-KO mice, and this effect is normalized soon afterward. This transient enhancement of NMDAR and AMPAR expression is accompanied by accelerated functional AMPAR responses that also return to WT levels in juvenile mice. These results support the idea that NR3A-containing NMDARs restrict the synaptic expression of NR1, NR2A, and GluR1. Because concentration of these glutamate receptor subunits and the development of AMPAR currents are key measures of synapse maturation, our data indicate that the absence of NR3A results in synapses that mature earlier than their WT counterparts. Such changes might be expected if, in the narrow temporal window of its highest expression, NR3A plays a regulatory role as a molecular brake for the functional and structural changes associated with synapse stabilization.

Our data suggest that NR3A levels not only decline with age, but NR3A also undergoes redistribution from the PSD to other biochemical compartments. In older animals, NR3A is relatively more abundant in the SPM fraction (which includes TSF and PSDs, Figure 2c: >P40) than in PSDs. This suggests that NR3A-containing receptors in adults may be preferentially targeted towards peri-/extrasynaptic sites. Such an age-dependent shift in subcellular location would be consistent with mature hippocampal culture data showing that NR3A-containing NMDARs have a relatively uniform distribution at the membrane surface, reflecting a weak association with the PSD [21]. In addition, a redistribution of NR3A away from synaptic sites may further limit synaptic activation of NR3A-containing NMDARs. Thus, NR3A-containing NMDARs on neurons may be rarely activated in adulthood, and this may occur only under highly specialized circumstances (such as periods of pronounced glutamate release and spillover).

We do not know whether increased synaptic localization of NR1, NR2A, and GluR1 in week-old NR3A-KO mice could be due to a change in transcription, translation, and/or a redistribution of glutamate receptors. This question will take additional experiments to answer, especially given that there is often discrepancy between transcript and protein levels [56]. We are intrigued by the possibility that the increased localization of NR1, NR2A, and GluR1 could be due to redistribution of these proteins into the PSD from non-synaptic pools. Glutamate receptors are present at both perisynaptic and extrasynaptic sites, diffusing laterally into the synapse under regulated conditions, presumably through stabilization by MAGUK scaffolding proteins and influenced by phosphorylation [57], [58], [59], [60], [61]. It is possible that NR3A-containing receptors, through direct actions or through interacting partners, may limit targeting of AMPARs and other NMDAR subtypes to the PSD.

It is not yet known how NR3A-containing receptors might be acting outside the PSD. Additional detailed ultrastructural analyses of NR3A localization [33], as well as biochemical studies that distinguish presynaptic and postsynaptic fractions [62], will provide insight into how NR3A-containing NMDARs are positioned to act outside the PSD. It is possible that the presence or absence of NR3A affects presynaptic functions, given that presynaptic NR3A-containing NMDARs have recently been shown to modulate neurotransmitter release and spike timing-dependent plasticity [33].

Intriguingly, NR3A directly suppresses NMDAR function [8], [12], [14] and can limit AMPAR-mediated synaptic currents (ref. [14], and this study). Because each dendritic spine is thought to contain a synapse [63], [64], and NR3A-KO mice have higher spine density [8], NR3A-KO mice would be expected to have more functional synapses. One possibility is that LTP-like synaptic enhancement in NR3A-KO mice might stabilize synapses, and this could account for the enhanced excitatory synaptic transmission we observed in young KO mice. Although increased neonatal AMPAR currents in NR3A-KO mice return to WT levels in juvenile animals, it is interesting that increased spine densities in NR3A-KOs remain elevated into adulthood [8]. Thus, the synaptic removal of NR3A may increase AMPAR currents at synapses, and this may help to stabilize synapses and lead to their persistence.

Synapses in many CNS regions undergo significant remodeling during postnatal brain development, although the molecules and signaling pathways responsible for the elimination of inappropriate synapses and the maintenance and strengthening of appropriate connections are still largely unknown [65], [66], [67], [68], [69], [70]. The role of NMDARs in regulating these processes is debated. Recent studies have separately proposed the ideas of NR2B and NR3A as negative regulators of synapse stabilization [14], [32], raising the question of whether NMDAR subunit composition and/or activation play a role. For example, because NMDAR assembly and insertion into forebrain synapses is thought to rely on a heteromeric combination of two NR1 subunits with NR3A and one NR2 subunit, the acceleration of synapse maturation induced by NR3A removal could be due to the selective loss of NR1/NR2B/NR3A triheteromers. The loss of NR2B function resulting in an increase in AMPAR-mediated mEPSC amplitudes [32] might also occur through the loss of not only the NR1/NR2B diheteromer, but also the NR1/NR2B/NR3A triheteromers, meaning that the NR2B-deficient mouse would effectively ablate both NR2B and NR3A expression.

NR3A expression at synapses is dependent on a number of factors, including its unique intracellular interactions and association with other NMDAR subunits. Unlike NR2 subunits [71], [72], PDZ binding sequences are conspicuously absent in NR3 subunits [11], [73]. Thus, the synaptic attachment of NR3A-NMDARs is critically dependent upon NR2A and/or NR2B that can be targeted to the PSD through PDZ-dependent interactions with membrane-associated guanylate kinases (e.g., PSD-95 and SAP102). Importantly, the synaptic removal of NR3A is an activity-dependent process [21]. However, the effects that MAGUK-NR2 interactions may have on the targeting, anchoring, and stabilization of NR3A at synaptic sites are unknown. Future experiments will be required to determine how the association between synaptic glutamate receptors and PSD scaffolding proteins changes during development. These will then provide further clues as to how NR3A-NMDARs act as a molecular brake by restricting the developmental onset of glutamate receptor expression. One clue may come from a recent report showing that a family of PDZ-binding domain proteins named takusans is upregulated in NR3A-KO animals, resulting in altered expression of AMPAR subunits to affect synaptic activity [74].

If NR3A is responsible for delaying the stabilization of NMDARs and the insertion of AMPARs at the synapse, the prolonged absence of NR3A would be expected to cause an increase in the fraction of mature synapses. This idea is consistent with the notion that activity-dependent NR3A removal by PACSIN may ‘unsilence’ synapses through the insertion of more mature NMDAR subtypes that trigger AMPAR insertion [14]. The early onsets of synaptic NMDAR currents and LTP in NR3A-KO mice [14] are also in agreement with this interpretation, although these differences are not maintained in P16 and adult mice. In this context, our data point to an as-yet-unidentified signaling mechanism that links NMDAR inhibition to restricted AMPAR trafficking. Alternatively, NR3A-containing receptors may be actively involved in mediating developmental synapse elimination. By limiting synapse potentiation, NR3A may affect the fundamental measures of synapse maturation: synapse size, synapse strength, and long-term memory.

Behavioral studies have only begun to address the long-term implications of NR3A in the refinement of neural circuits capable of learning and storing memories [14], [75]. Resolving the temporal and subcellular localization of NR3A protein, as we have done here, is an important step toward this goal. Taken together with recent data [14], our results contribute to a model whereby inclusion of NR3A in NMDARs alters connections in the brain anatomically, by influencing the number of dendritic spines, and functionally, through weakened synaptic connections. NR3A removal by PACSIN [21] would then relieve a developmental brake on excitatory synapse stabilization, thus allowing/facilitating the insertion of the more mature NMDAR subtype, NR2A, as well as AMPARs.
